# The Teeth

**Published:** 1882-01

**Authors:** 


**THE TEETH.**—Natural teeth, clean, sound and perfect, are essential to the comeliness of any human face. Defective teeth mar the handsomest features, and cause us to turn away our gaze with a kind of disgust from a countenance otherwise faultlessly beautiful. Sound teeth not only add to the comfort and personel appearance, but contribute largely to the health of all, hence special and scrupulous attention should be paid to them daily, from early childhood, from the time when the first permanent tooth makes its appearance, about the sixth year.

Whenever it is practicable, every tooth in a child's head should be minutely examined by a careful, conscientious, and skillful dentist every few months; and the great importance of special attention to their cleanliness, the avoidance of cold and hot drinks—of the use of any "picks" harder than wood or quills, and of all dentifrices prepared by unknown hands, should be impressed upon the minds of the young with great assiduity.

Harm has been done by propagating the notion that sugar is injurious to the teeth, by diverting attention from real causes of destruction or decay. The eating of any amount of pure sugar can not injure the teeth directly, because it has no residue, it is wholly dissolved and passes into the stomach.

But let it be remembered that the practice of eating sugars or candies or any other sweetmeats largely, will inevitably cause a disorder of the stomach and generate gases there, which will speedily undermine the health of the teeth.

By insisting too much on the fact that sugars and candies destroy the teeth, an impression will grow that if these are mainly avoided, the person so doing will have good teeth, and this leads the mind away from the necessity of keeping the mouth clean and the stomach healthful. If these things are well done, and the teeth are kept plugged in a finished style, teeth naturally or hereditarily "poor," may be kept in a good state of preservation for many years.

All forms of dyspepsia have a direct tendency to destroy the teeth. Whatever causes acidity of the stomach, when it is remembered how many patent medicines are made up in the form of syrups and sweet lozenges, and how common the use of them has become, it need not be wondered at that every second or third person met on the street knows the meaning of "sour stomach" or dyspepsia. It has been shown that if a sound tooth be steeped in syrup for some days, it becomes a soft, pulpy mass. That does not prove that syrup is injurious to the teeth, because it was a dead tooth; and further, such a steeping of a live tooth is impossible. The gastric juice is innocuous to a living stomach, but at the very moment of death, that same gastric juice begins to eat up the stomach. So it is inconclusive to reason from the living to the dead, or *vice versa.*

It is urged by many that calomel is a most deadly agent to the teeth, and yet if a sound tooth is soaked for weeks together in a solution of calomel, no apparent effect whatever is produced on it.

So far from sugars and pure candies injuring the teeth or the health, they would, if used wisely and in moderation, as sole desserts, be actual preventives of both; especially if alternated, as desserts, with fruits and berries in their natural, raw, ripe, fresh, perfect state, by banishing from our tables the pestiferous pie, the leaden pudding, and pastries and cakes of every name, which, as desserts, always tempt to excesses which lay the foundation for diseases which torture for a lifetime, or bring speedily to the grave.

Let the spirit of this article be distinctly understood. Pure sugars and candies do not injure the teeth, except indirectly, by their injudicious use in exciting acidity of stomach or dyspepsia, as will any other kind of food, or drink, or beverage, if extravagantly used.

At seasons of the year when fruits and berries may not be had, ripe, fresh, and perfect, as desserts, pure sugars and candies may be used as such in their stead to great advantage, because they are healthful, being warming, nutritious, and agreeable; hence, as a table article, they are very valuable, while the almost universal love of them shows that they were intended to be eaten. If a child is not allowed to eat any thing containing sugar it will sicken and die in a very short time. Children need the carbon, the fuel contained in sugar to keep them warm, without it they would perish from cold; hence the love of sweet things is an instinct, implanted by the kind and wise Maker of us all for the child's preservation. There are a parcel of stupid creatures in the world whose sole stock in trade of brains and logic amounts to this, that "whatever is good is unhealthy." It is not advised that children should be allowed to eat sugar and candy whenever they want it; but that as a dessert, after each regular meal, the use of pure sugars and candies would benefit and not injure.

A STATEMENT is going the rounds of the newspapers and scissored magazines without "credit," and thus without authority, which prevents an appreciating public, too lazy to think for themselves, the pleasure of knowing to whom they are indebted for an article so universally practical, and yet so perfectly convincing and logical in its statements and conclusions. It is headed:

"A NEW TOOTH-POWDER.—Roasted rye is recommended as tooth-powder, from the fact that, in all those countries where bread made of rye is the food of the generality of the inhabitants, the latter are remarkable for the whiteness, strength, and durability of their teeth. Savoy and Landes are instances of this truth. Schrader has found 500 grammes of ashes of rye to contain 7 grammes of carbonate of lime, 9.8 ditto of magnesia, 7.2 of oxyd of iron and manganese, and 1.9 of silica, all of which substances have a favorable effect on the teeth. Rye, finely pulverized and used daily as a tooth-powder, is said to stop caries, and promptly cure the small abscesses which are often formed on the gums."

This show of scientific terms will cause the statement to be received as perfectly conclusive by that large class of readers which voraciously gulp down as true all that is put in print, thus saving themselves the trouble of thinking. Reflecting persons, however, will call in question the legitimacy of the sequence that pulverized roasted rye, daily used as a tooth powder, will insure sound, white teeth, even if decay has already begun, because those who live on rye-bread have good teeth! It is this careless, incoherent, and illogical mixing up of the false and true, of mere theory with fact, which makes so many failures in life. Want of exactness in our knowledge is a striking characteristic of the American people. Knowledge is only practically valuable in proportion as it is exact. Hence those who "succeed in life" (with us "success" means making a large amount of money) rather blunder on a fortune than secure it by well-matured plans and operations. We might as well say that because taking water into the stomach satisfied thirst, washing the big toe would do the same thing.

It is beautifully and instructively true that the inhabitants of Savoy and Landes have strong, white, and durable teeth, but it does not necessarily follow that it is owing to the fact of their living on rye-bread. Perhaps it is because they do not eat much meat or do not drink whisky. May be it is because they do not drink much water in the south of France and in northern Italy, where wine is so abundant. Perhaps it is because they never read a daily newspaper, and thus are saved from burdening their memories and endangering their health and lives by multitudes of plausible but lying "receipts." But by a little scientific inquiry we may be able to decide with satisfactory conclusiveness whether there is any connection between eating rye-bread and having sound teeth. One familiar fact looking in that direction is, that certain soils will largely increase their yield of grain if lime is scattered over them. This proves that lime is a constituent of grains, and when a chemist analyzes them, he finds that lime *is* one of their largest constituents. If hens are fed on food which contains no lime, they will soon lay eggs without any shells, or will lay none at all; but give them lime to eat with their food, or give them food which has lime largely as a constituent, and the eggs will soon have shells on them. This would seem to show that egg-shells are mainly composed of lime, and such *is* the fact. As to the teeth, it is found that almost the entire bulk is lime. Some teeth are softer than others, and on examination it is found that they are soft because they have not the proper amount of lime in them.

Again, the "permanent" teeth of many of our children begin to decay before they are seven years old. On examination it is found that at some point, perhaps the center of the upper horizontal surface, there is a soft spot not covered with enamel, and it is because there was not lime enough in the system to complete the work of enameling the whole tooth—like some men who begin to build houses, and have to leave them unfinished for want of money to purchase materials to complete them. It would seem then that if grains are composed mainly of lime, and teeth wanting in whiteness, strength, and durability are deficient in lime, it is a rational inference that if grains were made the chief articles of food by a people, that people would have sound teeth. We feed on grains in the shape of bread. The main diet of the French is bread; and on our visit to the Louvre, in Paris, it was noticed, with considerable interest and wonder, that a young man or woman would paint until noon, then would make a hearty dinner on a loaf of bread and a bottle of wine, without leaving the apartment, and then paint on the remainder of the day. Persons were seen passing along the streets with loaves of bread on their shoulders a yard long. The fact is, the French make the best bread in the world, and on their bread and wine alone a man may live sumptuously every day. It very naturally follows then, that, as the main food of the French is made of grain, and Landes is in France, and the people of Landes have good teeth, and teeth are bad for want of lime, and grain (bread) has a great deal of lime in it, the way to give our children good teeth is to bring them up on bread. But that is taking rather a long jump, for we "fetch up all standing" against a stubborn little fact that all our children eat largely of bread, and yet dentists assure us that there are hundreds of children under ten years of age who have from one to half a dozen plugs of gold in their teeth. Is not the reader sorry to have such a beautiful theory spoiled, to say nothing of the trouble of hunting up another? But we must follow out the investigation. It never does any good to "give up," to get "discouraged," especially in matters of this kind; for this thing of having good teeth is one of prime importance in a variety of ways. It prevents dyspepsia; it adds to the pleasure and comfort of eating. Without good teeth the most splendid "figure" is nothing, and the handsomest face is not worth looking at. What woman would knowingly and willingly wed a man without "ivories"? The smile of beauty, how enrapturing! But if that smile displays a snaggled tooth, blackened and half-decayed, what a fall!—nothing less than fathomless! Young ladies, a fine set of pearly-white teeth (of your own!) will be of greater service to you in getting a good husband, a man of fine, elevated tastes, than the stocks, bonds, and mortgages of your father.

But so far from the grain and tooth theory being spoiled, a little further investigation will add both to its beauty and truthfulness. The people of Landes eat their bread with all the lime in it. We throw the lime away and eat the remainder! Landes and Savoy are out-of-the-way countries. The inhabitants are small farmers and poor, hence are careful, waste nothing, and prepare their food in the most primitive manner. In short, they eat the whole grain, either boiling it as we do rice or cracked wheat, or pound or grind it into coarse meal for bread, thus consuming the whole grain, husk, kernel, pith, heart, and all. It is true of grains as it is of the potato, the most nutritious and wholesome part is that immediately under the outer skin. The outer eighth of an inch of the potato contains more nourishment than all the remainder; hence it is a shameful waste to peel a potato with a knife, of which many of the poor are unfortunately ignorant to their own great loss. Thus it is that the outer portion of ground grain, called the "bran," is richest in nutriment and contains nearly all the lime; but by refining it away, in our efforts to get a "fine" and "white" flour, we but eat the refuse and throw away the substance, and thus lose the lime which gives strength to the bones, durability to the teeth, and vigor to the brain, through the pure, perfect, and life-giving blood which the consumption of the whole grain makes.

In point of physical vigor and development it would be of incalculable value to our country if the children were allowed to take nothing for their breakfast and supper, as their general habit, until the twelfth year is completed, but milk with mush, cracked wheat, porridge, or other forms of food which include the entire grain.

There is perhaps no one living whose bowels are not made free or costive by particular articles of food; the same article affects different persons variously. Each man must therefore observe for himself what articles constipate, and what loosen and act accordingly; a world of suffering and multitudes of lives would be saved every year by a proper attention to this simple suggestion, but not one man or woman in a thousand will give it that attention, hence the great mass of humanity perishes before its prime.

There are some articles of food which have various effects according to the parts used. The *May Apple*, or "Mandrake," is a nutritious fruit; its root is cathartic, its leaves a poison. The common house grape is a luscious product: the pulp is a delicious food, and in health should be the only part swallowed; the seeds loosen the bowels, while the skin constipates them. Two or three pounds of freshly picked, ripe grapes, may be eaten daily by a person in good health. The best time for eating them is immediately after breakfast and dinner.

The only safe, as well as the most rational practice of physic, is to make our food subserve medical uses. Knowing this, a doctor no more takes his own pills than an attorney goes to law, or a divine practises his own preaching

